# The prognostic significance of RUNX2 and miR-10a/10b and their inter-relationship in breast cancer

**DOI:** 10.1186/s12967-014-0257-3

**Published:** 2014-09-30

**Authors:** Chih-Hao Chang, Tan-Chi Fan, Jyh-Cherng Yu, Guo-Shiou Liao, You-Chin Lin, Arthur Chun-Chieh Shih, Wen-Hsiung Li, Alice Lin-Tsing Yu

**Affiliations:** Graduate Institute of Life Sciences, National Defense Medical Center, Taipei, 114 Taiwan; Institute of Stem Cell and Translational Cancer Research, Chang Gung Memorial Hospital at Linkuo & Chang Gung University, No.5, Fu-Shin St., Kuei Shang Taoyuan 333, Taoyuan, Taiwan; General Surgery, Department of Surgery, Tri-Service General Hospital, National Defense Medical Center, Taipei, 114 Taiwan; OPKO Taiwan Incorporation, Hsinchu County 302, Hsinchu, Taiwan; Institute of Information Science, Academia Sinica, Taipei, 115 Taiwan; Biodiversity Research Center, Academia Sinica, 128 Academia Road Sec. 2, Nankang Taipei, Taipei, 115 Taiwan; Department of Ecology and Evolution, University of Chicago, Chicago, IL 60637 USA; Department of Pediatrics, University of California in San Diego, San Diego, CA 92103 USA

**Keywords:** RUNX2, miR-10a, miR-10b, Breast cancer prognosis

## Abstract

**Background:**

The major cancer related mortality is caused by metastasis and invasion. It is important to identify genes regulating metastasis and invasion in order to curtail metastatic spread of cancer cells.

**Methods:**

This study investigated the association between RUNX2 and miR-10a/miR-10b and the risk of breast cancer relapse. Expression levels of RUNX2 and miR-10a/b in108 pairs of tumor and non-tumor tissue of breast cancer were assayed by quantitative PCR analysis and evaluated for their prognostic implications.

**Results:**

The median expression levels of RUNX2 and miR-10b in tumor tissue normalized using adjacent non-tumor tissue were significantly higher in relapsed patients than in relapse-free patients. Higher expression of these three genes were significantly correlated with the hazard ratio for breast cancer recurrence (RUNX2: 3.02, 95% CI = 1.50 ~ 6.07; miR-10a: 2.31, 95% CI = 1.00 ~ 5.32; miR-10b: 3.96, 95% CI = 1.21 ~ 12.98). The joint effect of higher expression of all three genes was associated with a hazard ratio of 12.37 (95% CI = 1.62 ~ 94.55) for relapse. In a breast cancer cell line, RUNX2 silencing reduced the expression of miR-10a/b and also impaired cell motility, while RUNX2 overexpression elicited opposite effects.

**Conclusions:**

These findings indicate that higher expression of RUNX2 and miR-10a/b was associated with adverse outcome of breast cancer. Expression levels of RUNX2 and miR-10a/b individually or jointly are potential prognostic factors for predicting breast cancer recurrence. Data from *in vitro* studies support the notion that RUNX2 promoted cell motility by upregulating miR-10a/b.

**Electronic supplementary material:**

The online version of this article (doi:10.1186/s12967-014-0257-3) contains supplementary material, which is available to authorized users.

## Background

Breast cancer is the most common malignant disease in women. Approximately 10 ~ 15% breast cancer patients have an aggressive disease and develop distant metastases [1] to bone, lung, liver and brain [2]. Up to 70% of bone metastases occur in advanced breast cancer patients [3]. Moreover, five year survivability for breast cancer patients with bone metastasis is only 20% after its detection [4].

Runt-related transcription factor 2 (RUNX2) has been shown to play an important role in osteogenesis and development of osteosarcoma [5]. Runx2 was also reported to be highly expressed in breast cancer with poor clinical outcomes [6]. Recently, McDonald *et al.* showed that high RUNX2 expression is significantly associated with estrogen receptor (ER)/progesterone receptor (PR)/HER2-negative breast cancers and that patients with high RUNX2 expression have a poorer survival rate than those with negative or low expression [7]. Moreover, in non-small cell lung cancer-patients, higher RUNX2 expression was significantly correlated with tumor progression and metastasis [8]. In epithelial ovarian cancer, various genes involved in tumor invasion and metastasis were suppressed upon RUNX2 knockdown [9]. Studies of breast cancer revealed regulation of several genes involved in bone invasion, such as MMPs, VEGF, OP, and BSP, by RUNX2, suggesting that this master transcription factor might contribute to bone metastasis in breast tumor [10-13]. This is consistent with the report that RUNX2 silencing reduced cell motility of metastatic breast cancer cell line, MDA-MB-231. On the other hand, RUNX2 overexpression increased cell migration ability in non-metastatic MCF7 breast cancer cell line [14].

MicroRNAs (miRNAs), a group of ~22 nucleotides endogenous and evolutionarily conserved single-stranded small non-coding RNAs, are crucial post-transcriptional regulators of a variety of biological processes, including the initiation, progression and metastases of cancer [15-18]. As reported in several studies, the miRNA-10 (miR-10) family, including miR-10a and miR-10b which are identical except for the 12^th^ nucleotide [19], play an important role in tumorigenesis and progression [20,21]. MiR-10a was reported to be downregulated in chronic myeloid leukaemia and acute myeloid leukaemia, and upregulated in colon cancer and hepatocellular carcinomas [20]. Mir-10a was also reported to gain in copy number in melanoma and breast cancer [22] and overexpression of miR-10a promoted cell migration and invasion of hepatoma cancer cell lines [23] and cervical cancer cell lines *in vitro* [24]. On the other hand, miR-10b was upregulated in pancreatic cancer and B-cell chronic lymphocytic leukemia [20] In addition, miR-10b was highly expressed in breast tumor with poor clinical outcomes [25] and facilitated cell migration and invasion in breast cancer [26].

These findings suggest that RUNX2 and miR-10a/b play important role in progression and metastases in breast cancer, but the association between RUNX2 and miR-10a/b, if any, is unknown. In this study, we try to decipher the relationship between RUNX2 and miR-10a/b in clinical breast cancer samples as well as in cell lines. We demonstrated that expression of RUNX2 significantly correlated with miR-10a/b in ER negative and triple negative breast cancers and the expression levels of RUNX2 and miR-10a/b individually or jointly were significant prognostic factors for predicting breast cancer recurrence. Furthermore, RUNX2 silencing in MDA-MB-231 cells downregulated miR-10a/b transcription and clearly impeded cell motility. These results indicated that RUNX2 plays an important role in regulating breast cancer progression.

## Methods

### Study patients and tissues

Ninety five of the 108 breast cancer patients examined in this study had clinicopathologically confirmed primary ductal carcinoma of the breast, and the remaining 13 patients had non-ductal carcinoma of the breast. All of them were diagnosed at the Tri-Service General Hospital, Taipei, Taiwan between October 1994 and February 2013. Patients’ clinical information, including cancer stage, tumor grade, estrogen receptor (ER), progesterone receptor (PR), HER-2/neu, recurrence and survival status, were also noted. Recurrent breast tumors were subjected to pathological confirmation to exclude the possibility of second primary tumors. Moreover, the cause of death was verified from death certificate; patients whose death was clearly documented to be due to breast cancer were considered to have died of breast cancer, whereas other causes of deaths were considered censored events. Tumor and adjacent non-tumor breast tissues from patients were obtained at the time of initial surgery, and were fully encoded to protect patient confidentiality. Clinical specimens were utilized under a protocol approved by the Institutional Review Board of the Human Subjects Research Ethics Committee of Academia Sinica and Tri-Service General Hospital, Taipei, Taiwan.

### Statistical analysis

Histogram data were given as mean ± standard deviation (SD). Comparison between the tumor and non-tumor breast tissues was performed using Student’s t-test. We examined whether there was any correlation between the expression levels of the miR-10a/b and RUNX2 genes, individually or jointly, in primary cancer tissue and the clinicopathological features of the tumor. The goal was to evaluate the usefulness of these three genes as prognostic biomarkers for breast cancer. The expression levels of the three genes in tumor or non-tumor tissues of different clinicopathological feathers were described by mean ± SD and median. The results of statistical analyses were examined against those of Student’s t-test and Wilcoxon rank-sum test. The estimated areas under the curves (AUC) for assessing the accuracy of prediction were calculated based on the receiver operating characteristic (ROC) curves, where an optimal diagnostic cutoff point was determined to differentiate over expression of individual genes. Based on these cutoffs, quantitative measurements of the expression levels of individual genes were converted into binary measurements to help examine whether over expression of these genes could be prospectively associated with breast cancer progression using the relapse-free survival (RFS) and overall survival (OS) as outcomes of interest. RFS was measured as the time from surgery to recurrence or the end of the study, and OS was defined as the time from surgery to death or the end of the last follow-up for a patient. Survival curves were plotted by using the Kaplan–Meier method and 5-year RFS-rate and 10-year OS-rate point estimates with 95% confidence interval (CI) were reported. The significance of these associations was assessed using the log-rank test. The Cox regression model was used to compute hazard ratios. *P*-value less than 0.05 were considered statistically significant. All data analyses were performed using the SAS statistical software for Windows, version 9.3 (SAS institute, Cary, NC, USA).

### Cell cultures

MCF7 breast cancer cell line was cultured in Modified Eagle Medium (MEM) supplemented with 10% fetal bovine serum (FBS). MDA-MB-231 and MB157 breast cancer cell lines, U2OS osteosarcoma cell line and 293 T embryonic kidney were cultured in Dulbecco’s Modified Eagle Medium (DMEM) supplemented with 10% FBS. BT483 breast cancer cell line was cultured in Roswell Park Memorial Institute medium (RPMI) supplemented with 10% FBS. All cell lines were obtained from American Type Culture Collection (ATCC, Manassas, VA, USA).

### RUNX2 plasmid, RUNX2 siRNA and miRNA Oligo synthesis and transfection

Total RNA from the osteosarcoma cell line U2OS was extracted using Trizol reagent (Invitrogen, Carlsbad, CA, USA), and 1 μg of RNA was reverse transcribed using the SuperScript III RT–PCR kit (Invitrogen). For cDNA cloning, full-length RUNX2wt was amplified from cDNA of the U2OS cell line using the PCR primer pair (RUNX2wt-forward: 5′-GCTAGCATGGACTACAAAGACGATGACGACAAGGTGATGCGTATTCCCGTAGATCCGAG-3′) and (RUNX2wt-reverse:5′-CTCGAGATATGGTCGCCAAACAGATTCATCC-3′) in an amplification mixture containing 1X *Pfu* polymerase buffer (Stratagene, La Jolla, CA, USA), 100 μM 4dNTPs, 1.5 mM MgCl_2_ and 1U *Pfu* polymerase (Stratagene). The samples were amplified for 35 cycles of 95°C for 1 min 30 sec, 60°C for 35 sec, and 72°C for 2 min each, preceded by a 5 min denaturation at 95°C and followed by a 10 min extension at 72°C. The amplified cDNAs of RUNX2wt were then cloned into pcDNA3.1 (+) vector (Invitrogen) by ligation of the *NheI* – *XhoI* fragments to generate the pcDNA-RUNX2 expression constructs. The sequence of pcDNA-RUNX2 was confirmed by DNA sequencing.

RUNX2 siRNA (*Cat. #*1027020) and siRNA control (*Cat. #*1027280) were guaranteed products by manufacturers and purchased from Qiagen (Valencia, CA, USA). Precursor miRNA oligos (pre-miR control *#*1: *Cat. #*AM17110, pre-has-miR-10a: *Cat. #*PM10787 and pre-has-miR-10b: *Cat. #*PM11108) and anti-miRNA oligos (anti-miR control *#*1: *Cat. #*AM17010, anti-has-miR-10a: *Cat. #*AM10787 and anti-has-miR-10b-5p: *Cat. #*AM11108) were guaranteed products by manufacturers and purchased from Ambion (Austin, TX, USA).

For plasmid transfection, 4 g of pcDNA-RUNX2 construct plasmid per well with 4 μl Lipofectamine 2000 (Invitrogen) was transfected into cell lines. For siRNA transfection, 25 nM RUNX2 siRNA per well with 2 μl Lipofectamine RNAiMax (Invitrogen) was transfected into cell lines. For miRNA oligos transfection, 25 nM miRNA oligos per well with 2 μl RNAiMax were transfected into cell lines. For anti-miRNA oligo transfection, 15 nM anti-miRNA oligos per well with 2 μl RNAiMax were transfected into cell lines. The experiments were repeated three times using six-well plates.

### Real-time PCR quantification of miRNA-10a/miR-10b and RUNX2

Total RNAs were extracted from tumor and non-tumor breast tissues for each patient or breast cancer cell lines using Trizol reagent according to the manufacturer’s specifications. 4 ng of total RNAs were used for quantification of miRNAs expression, including miRNA-10a/miR-10b and RNU6B (U6) RNA, by TaqMan RT-qPCR kit (miRNA-10a: *Cat. #*000387, miRNA-10b: *Cat. #*002218, and RNU6B: *Cat. #*001093; Applied Biosystems, Foster City, CA, USA) according to the manufacturer’s instructions. For the quantification of mRNA of RUNX2 and GAPDH, 1 g of total RNAs was reverse transcribed into cDNA using the SuperScript III kit and the specific mRNAs were detected by Power SYBR Green PCR Master Mix (Applied Biosystems) according to the manufacturer’s instructions. The primer sequences of RUNX2 and GAPDH qPCR were: RUNX2 5′-AGCAAGGTTCAACGATCTGAGATT-3′ (forward) and 5′-AAGACGGTTATGGTCAAGGTGAAA-3′ (reverse); GAPDH 5′-CTGCTCCTCCTGTT CGACAGT-3′ (forward) and 5′-ACCTTCCCCATGGTGTCTGA-3′ (reverse). The gene expression was normalized based on threshold cycle (Ct) values of common internal control for miRNA quantification assays, U6 RNA, and RUNX2 quantification assays, GAPDH. The level of RUNX2 and miR-10a/b expression were measured using the 2^-△△Ct^ method [27]. The results were presented as fold change of each gene in the tumor tissue relative to the non-tumor tissue. The RT-qPCR was performed on the 7300 Real-Time PCR System (Applied Biosystems). The procedures were repeated three times for all samples, and mean values were obtained for statistical analysis.

### Western blotting

Cell lysates were extracted in 0.1 M NaCl, 0.01 M TriszHCl (pH 7.6), 1 mM EDTA (pH 8.0), 1 mg/ml aprotinin, and 100 mg/ml PMSF, while protein concentration was determined by a Bio-Rad protein assay. Protein (50 μg) was boiled at 95°C in sodium dodecyl sulfate (SDS) sample buffer for 5 min, electrophoresed on 7–12% SDS-PAGE gels, and transferred to polyvinyldifluoridine membranes. These were then incubated overnight at 4°C with (*i*) rabbit monoclonal antibody against human RUNX2 (Cell Signaling Technology, Danvers, MA, USA; #8486; 1:250), (*ii*) goat polyclonal antibody against human HOXA1 (R&D Systems Inc., Minneapolis, MN, USA; AF5014; 1:500), (*iii*) goat polyclonal antibody against human HOXD10 (Santa Cruz Biotechnology, Dallas, TX, USA; sc-33005; 1:500), or (*vi*) rabbit polyclonal antibody against human Actin (Santa Cruz Biotechnology; sc-1615-R; 1:3000). Membranes were washed three times for 15 min with phosphate buffered saline (PBS) containing 0.1% Tween20 (PBST), incubated with anti-goat or anti-rabbit secondary antibody (both Santa Cruz Biotechnology; 1:5000) at room temperature for 60 min, and then washed three times for 15 min with PBST. The signals of protein bands were visualized using ECF western blotting kit (Amersham Biosciences, Piscataway, NJ, USA) and detected by Typhoon 9400 imager (Amersham Biosciences).

### Cell migration and invasion assays

Cell migration and invasion were assayed using a transwell chamber (Millipore, Temecula, CA, USA) with or without Matrigel (BD, Franklin Lakes, NJ, USA). 1 ml serum free medium was added into a 24-well plate, and a transwell chamber was placed into the well in both assays. The chamber was coated with 50 μl Matrigel and incubated for 2 hours at 37°C for the invasion assay. After transfection for 72 hours, in migration assay: cells were trypsinized and seeded into chambers at the density of 1 × 10^4^ cells for MDA-MB-231 and 3 × 10^4^ cells for MCF7 per well; and in invasion assay: cells were 2 × 10^4^ cells for MDA-MB-231 and 6 × 10^4^ cells for MCF7. Eight hours later for migration and 24 hours later for invasion in MDA-MB-231, and 12 hours later for migration and 32 hours later for invasion in MCF7, migrated cells remaining in the basal chamber were fixed and stained with 10% ethanol in 1% crystal violet for 30 min and washed three times with PBS. Non-migrated cells were removed by cotton swabs. Images of the stained cells were obtained under a phase-contrast microscope (Olympus, Tokyo, Japan). To quantify the number of invading cells, crystal violet-stained cells in visual fields were counted. The experiments were repeated three times and mean values were obtained for statistical analysis. Detailed materials and methods can be found in Additional file 1.

## Results

### Clinical features of breast cancer patients

It has been shown that RUNX2 plays an important role in cell migration and invasion in breast cancer and higher expression is associated with poor outcome [6,8,14]. Meanwhile, others have reported the contribution of miR-10a/b to cell migration and invasion in breast cancer and miR-10b overexpression correlates with poor prognosis [23-26]. However, the inter-relationship between RUNX2 and miR-10a/b and their joint impact on prognosis in breast cancer has yet to be investigated. We examined one hundred and eight patients with histologically proven breast cancers to delineate the relationship between the expression of RUNX2 and miR-10a/b, and clinical outcomes. Their clinical and pathological features were summarized in Table 1. The mean age was 59.2 ± 13.2 years and the median follow up for relapsed patients and overall survival was 54.6 and 63.2 months, respectively. The distribution of cancer stages was stage 0 (2.80%), stage I (31.78%), stage II (42.99%) and stage III (22.43%), respectively, and of the molecular subtypes was Luminal A (ER + or PR+, Her2-) (33.33%), Luminal B (ER + or PR+, Her2+) (35.19%), Her2 positive (ER-, PR-, Her2+) (15.74%) and triple negative (ER-, PR-, Her2-) (15.74%), respectively. These clinical and pathological characteristics of the breast cancer subjects across the strata of age, hormone receptor status, and Her2, were similar to those reported in other breast cancer clinics in Taiwan [28-30].

**Table 1:** **Clinical and pathological characteristics of breast cancer patients**

**Characteristics** ^**a**^	**N (%)**
**Age (mean ± SD and range)**	**59.2 ± 13.2 (35–102 yrs)**
**Gender**	
**Male**	1 (0.9%)
**Female**	107 (99.1%)
**Histology**	
**Ductal**	95 (88.0%)
**Other**	13 (12.0%)
**Grade**	
**I**	7 (6.6%)
**II**	36 (34.0%)
**III**	63 (59.4%)
**Stage**	
**0**	3 (2.8%)
**I**	34 (31.8%)
**II**	46 (43.0%)
**III**	24 (22.4%)
**ER**	
**Negative**	48 (44.4%)
**Positive**	60 (55.6%)
**PR**	
**Negative**	40 (37.0%)
**Positive**	68 (63.0%)
**Her2**	
**Negative**	53 (49.1%)
**Positive**	55 (50.9%)
**Molecular subtype**	
**Luminal A**	36 (33.3%)
**Luminal B**	38 (35.3%)
**ER-, PR-, Her2+**	17 (15.7%)
**ER-, PR-, Her2-**	17 (15.7%)
**Regional lymph node involvement**	
**No**	62 (58.5%)
**Yes**	44 (41.5%)
**Relapse**	
**No**	71 (65.7%)
**Yes**	37 (34.3%)
**RFS duration median (range)**	54.6 (1.1 ~ 100.9 months)
**Outcomes**	
**Alive**	100 (92.5%)
**Dead**	8 (7.5%)
**OS duration median (range)**	63.2 (1.6 ~ 217.9 months)

ER: estrogen receptor, PR: progesterone receptor, HER2: HER2/neu, RFS: relapse free survival, OS: overall survival.

Status of ER and PR was classified as “Positive” when ≧10% of cells were stained; HER2 status was defined as “Positive” when Dako score was 2+ or 3 + .

^a^Clinical characteristics according to the sixth edition of the AJCC Cancer Staging Manual (2006).

### The relationship between expression of RUNX2 and miR-10a/b genes in breast cancer tumors and clinical parameters

The expression levels of RUNX2 and miR-10a/b were determined in tumor tissues and adjacent non-tumor tissues by qPCR. As shown in Table 2, the median expression levels of RUNX2 and miR-10b were significantly higher in tumor tissue than in non-tumor tissues by 2.16 and 1.73 fold, respectively (p < 0.0001, p < 0.0001). There is also a trend for higher expression of miR-10a in tumor tissues than in non-tumor part, but the difference did not reach statistical significance (p = 0.1318). Patients with ER- or triple negative subtypes displayed higher-level expression of these three genes than those with ER + or non-triple negative subtypes. Analysis of clinical outcome revealed that patients who relapsed or died of breast cancer showed higher expression of these three genes than non-relapse or alive patients. On the other hand, no significant association was found between the expression levels of these three genes and gender, histology, stage, regional lymph node involvement or status of PR and Her2, although there was a trend for higher expression in patients with higher histology grade (Table 2; Additional file 1: Table S1).

**Table 2:** **Correlation of expression of miR-10a, miR-10b and RUNX2 with clinical parameters of breast cancer patients**

	**miR-10a**				**miR-10b**				**RUNX2**			
**Variable**	**Mean ± SD**	**Median (Range)**	**P value** ^**a**^	**P value** ^**b**^	**Mean ± SD**	**Median (Range)**	**P value** ^**a**^	**P value** ^**b**^	**Mean ± SD**	**Median (Range)**	**P value** ^**a**^	**P value** ^**b**^
**Tissue**												
**Non-tumor**	1.00 ± 0.00	1.00 (1.00 ~ 1.00)	Ref.	Ref.	1.00 ± 0.00	1.00 (1.00 ~ 1.00)	Ref.	Ref.	1.00 ± 0.00	1.00 (1.00 ~ 1.00)	Ref.	Ref.
**Tumor**	2.02 ± 0.28	1.19 (0.01 ~ 18.57)	**0.0003** ^**c**^	0.1318^d^	3.13 ± 0.42	1.73 (0.07 ~ 26.72)	**<0.0001** ^**c**^	**<0.0001** ^**d**^	522.43 ± 381.75	2.16 (0.01 ~ 37902.36)	0.1748^c^	**<0.0001** ^**d**^
**ER**												
**Negative**	2.88 ± 0.53	1.61 (0.07 ~ 18.57)	Ref.	Ref.	4.32 ± 0.76	2.24 (0.08 ~ 26.72)	Ref.	Ref.	818.84 ± 789.26	3.64 (0.53 ~ 27902.36)	Ref.	Ref.
**Positive**	1.33 ± 0.23	0.86 (0.01 ~ 11.91)	**0.0085**	**0.0026**	2.18 ± 0.40	1.49 (0.07 ~ 20.34)	**0.0148**	**0.0134**	285.31 ± 276.77	1.43 (0.01 ~ 16612.71)	0.5260	**<0.0001**
**Molecular subtypes**												
**Luminal A**	1.34 ± 0.25	0.84 (0.04 ~ 7.48)	**0.0178**	**0.0012**	2.16 ± 0.31	1.54 (0.11 ~ 6.68)	**0.0303**	**0.0064**	8.29 ± 6.45	1.47 (0.01 ~ 233.94)	0.8339	**<0.0001**
**Luminal B**	1.97 ± 0.41	1.35 (0.01 ~ 11.91)	0.0501	**0.0305**	3.22 ± 0.76	1.87 (0.07 ~ 20.34)	0.0965	**0.0146**	1466.29 ± 1077.31	2.02 (0.30 ~ 37902.36)	0.1846	**0.0070**
**ER-, PR-, Her2+**	1.96 ± 1.06	0.88 (0.07 ~ 18.57)	0.2294	**0.0047**	2.34 ± 0.98	0.92 (0.08 ~ 15.79)	0.0637	**0.0067**	14.06 ± 7.34	2.72 (0.54 ~ 127.12)	0.5935	0.2280
**ER-, PR-, Her2-**	3.63 ± 0.85	2.10 (0.37 ~ 13.55)	Ref.	Ref.	5.80 ± 1.51	5.49 (0.33 ~ 26.72)	Ref.	Ref.	9.78 ± 2.89	5.94 (1.17 ~ 50.56)	Ref.	Ref.
**Relapse**												
**No**	1.83 ± 0.34	1.02 (0.04 ~ 18.57)	Ref.	Ref.	2.46 ± 0.40	1.36 (0.07 ~ 20.34)	Ref.	Ref.	5.53 ± 1.85	1.88 (0.26 ~ 127.12)	Ref.	Ref.
**Yes**	2.37 ± 0.48	1.35 (0.01 ~ 13.55)	0.3753	0.1002	4.42 ± 0.91	2.04 (0.08 ~ 26.72)	0.0578	**0.0171**	1514.33 ± 1105.78	3.46 (0.01 ~ 37902.36)	0.1809	**0.0268**
**Outcomes**												
**Alive**	1.86 ± 0.27	1.10 (0.01 ~ 18.57)	Ref.	Ref.	2.92 ± 0.42	1.55 (0.07 ~ 26.72)	Ref.	Ref.	184.40 ± 166.24	2.07 (0.01 ~ 16612.71)	Ref.	Ref.
**Dead**	3.96 ± 1.32	2.03 (0.84 ~ 10.56)	**0.0450**	**0.0270**	5.77 ± 1.84	5.14 (1.91 ~ 17.88)	0.0723	**0.0099**	4747.89 ± 4736.36	7.02 (0.30 ~ 37902.36)	0.3676	0.1017

ER: estrogen receptor, PR: progesterone receptor, HER2: HER2/neu, Ref.: reference group.

The expression level of the indicated gene in tumor tissue as determined by qPCR was normalized to that of adjacent non-tumor tissue of the same patient (2^-△△Ct^) (see Methods for details).

^a^P value for independent t-test.

^b^P value for Wilcoxon rank-sum test.

^c^P value for paired t-test.

^d^P value for Wilcoxon signed-rank test.

Bold face: statistical significant (P value < 0.05).

### Overexpression of RUNX2 and miR-10a/b genes in tumors as predictors of breast cancer outcome

To evaluate the prognostic significance of expression levels of RUNX2 and miR-10a/b, we used the receiver operating characteristic (ROC) curves based on the expression levels of RUNX2 and miR-10a/b in individual tumor/non-tumor ratios to determine the cutoff values defining “overexpression” of the three genes. According to these cutoff values, the areas under the curves (AUC) values for individual or various combinations of these three genes were found to be significantly lower in non-relapsed patients than in relapsed patients. However, when comparing patients with fatal outcome to those alive, only AUC values for miR-10b and combination of these three genes were significantly lower in alive patients than in those who died (Additional file 1: Table S2). Accordingly, we used the cutoff values to define the status of “overexpression” in subsequent logistic and COX regression analyses to ascertain correlations between gene expression and prognostic features. Under these cutoff values, the frequencies of overexpression of RUNX2 and miR-10a/b genes were significantly higher in patients who relapsed, and that of miR-10b overexpression was higher in patients who died (all p < 0.05). Using the same dichotomized values for logistic regression analysis, the odds ratios (ORs) for relapse were higher in patients with increased mRNA or miRNA levels of these 3 individual genes, while the OR for death was only associated with miR-10b overexpression (all p < 0.05). Patients with no overexpression of these three genes served as the reference group. In terms of the joint effects of overexpression of all three genes on prognosis, the ORs were respectively 20.32 (95% confidence interval (CI) = 1.50 ~ 179.92) and 17.05 (95% CI = 1.76 ~ 165.01) for relapse and death (P < 0.05). There was a trend toward an incremental additive effect on significantly elevated risk of relapse (OR = 2.66; 95% CI = 1.50 ~ 4.75), death (OR = 2.90; 95% CI = 1.22 ~ 6.89) and regional lymph node involvement (OR = 1.51: 95% CI = 1.03 ~ 2.21), respectively, as patients carried a greater number of overexpressed genes (P-trend < 0.05; Table 3; Additional file 1: Table S3).

**Table 3:** **The relationship between overexpression of individual gene or joint effects of miR-10a, miR-10b, and RUNX2 with prognosis in breast cancer patients**

	**Relapse**					
	**No**	**Yes**				
**Variable**	**Number (%)**	**Number (%)**	**P-value**	**OR**	**95% CI**	**P value**
**miR-10a**			**0.0424**			
**≦0.53**	27 (79.4%)	7 (20.6%)		1.00		
**>0.53**	44 (59.5%)	30 (40.5%)		2.83	1.05 ~ 7.68	**0.0407**
**miR-10b**			**0.0043**			
**≦0.67**	24 (88.9%)	3 (11.1%)		1.00		
**>0.67**	47 (58.0%)	34 (42.0%)		5.37	1.46 ~ 19.81	**0.0116**
**RUNX2**			**0.0037**			
**≦3.05**	51 (76.1%)	16 (23.9%)		1.00		
**>3.05**	20 (48.8%)	21 (51.2%)		3.55	1.47 ~ 8.58	**0.0048**
**Number of overexpressing genes**			**0.0021**			
**0**	16 (94.9%)	1 ( 5.8%)		1.00		
**1**	11 (73.3%)	4 (26.7%)		3.30	0.30 ~ 36.29	0.3288
**2**	32 (68.1%)	15 (31.9%)		6.01	0.71 ~ 50.83	0.0996
**3**	12 (41.4%)	17 (58.6%)		20.32	2.30 ~ 179.92	**0.0068**
**Additive model of gene overexpression**				2.66	1.50 ~ 4.75	**0.0009**

OR: odds ratio, CI: confidence interval, Gene overexpression: the expression level of an individual gene in tumor tissue normalized to adjacent non-tumor tissue from the same patient was categorized as “overexpression” by using the cutoff value determined from the ROC curve.

ORs and 95% CIs were estimated in the logistic regression model, in which a group of dummy variables was used to represent different groups of patients showing different numbers of overexpressing genes.

Bold face: statistical significant (P value < 0.05).

### The correlations between overexpression of RUNX2 and miR-10a/b and poor clinical outcome

**Figure 1:**
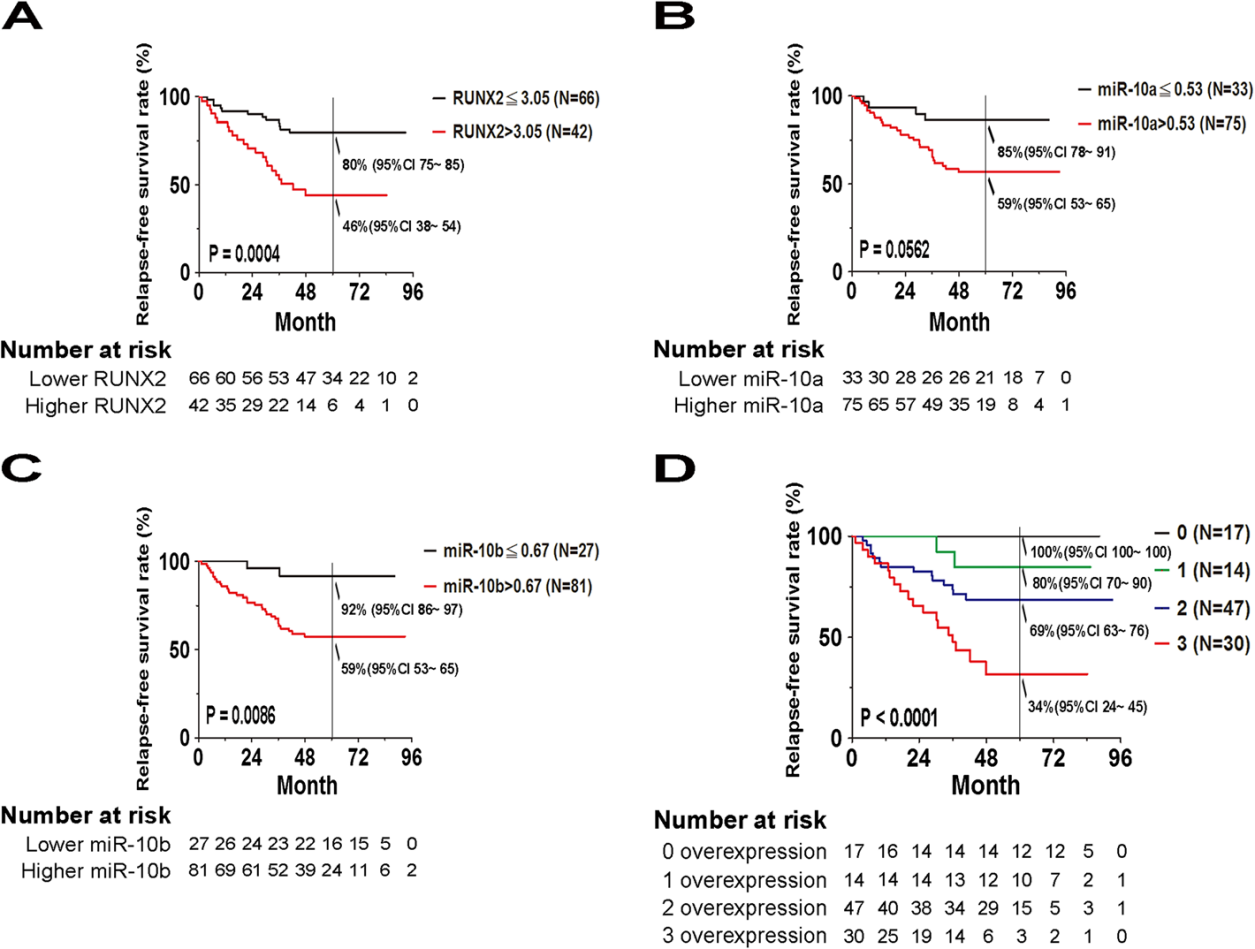
**Prognostic significance of increased expression levels of RUNX2, miR-10a or miR-10b in breast cancer patients.** Kaplan-Meier statistical analyses were conducted to examine the association between relapse-free survival and the expression of RUNX2 **(A)**, miR-10a **(B)** or miR-10b **(C)**, and the number of overexpressed genes **(D)** in all patients.

The correlations between overexpression of RUNX2 and miR-10a/b, individually or jointly, and clinical outcomes were examined by the Kaplan–Meier method of patients. Both RFS (Figure 1) and OS (Additional file 1: Figure S1) were significantly lower in patients with overexpression of RUNX2 or miR-10b individually (Figure 1A, C; Additional file 1: Figure S1 A, C). Compared to the patients with no overexpression of these three genes, those with a greater number of overexpressed genes (1, 2, or 3) in tumors showed lower rates of RFS (log-rank p < 0.0001) and OS (log-rank p = 0.0002) (Figure 1D and Additional file 1: Figure S1D). Multivariate Cox regression analysis showed a significant correlation between higher hazard ratios (HRs) for relapse and greater gene signals for all three genes individually and a correlation between higher HR for death and expression of miR-10b (Table 4; Additional file 1: Table S4). As to the joint effects of overexpression of all three genes compared with no overexpression of any of them on prognosis, the HRs were 12.37 (95% CI = 1.62 ~ 94.55) and 14.59 (95% CI = 1.66 ~ 128.24) for relapse and death, respectively (P = 0.015, 0.016). The multiple gene additive model displayed a statistically significant HR of 2.25 for RFS (95% CI = 1.44 ~ 3.52, P-trend = 0.0004) and 2.88 for OS (95% CI = 1.26 ~ 6.61, P-trend = 0.0125).

**Table 4:** **Cox regression model analyses of the gene expression levels in association with RFS of breast cancer patients**

	**RFS**		
**Variable**	**HR**	**95% CI**	**P value**
**miR-10a > 0.53 V.S miR-10a≦0.53**	2.31	1.00 ~ 5.32	0.0503
**miR-10b > 0.67 V.S miR-10b≦0.67**	3.96	1.21 ~ 12.98	**0.0231**
**RUNX2 > 3.05 V.S RUNX2≦3.05**	3.02	1.50 ~ 6.07	**0.0020**
**Number of overexpressing genes**			
**0**	1.00		
**1**	2.80	0.30 ~ 25.77	0.3642
**2**	4.99	0.65 ~ 38.12	0.1212
**3**	12.37	1.62 ~ 94.55	**0.0153**
**Additive model of gene overexpression**	2.25	1.44 ~ 3.52	**0.0004**

Adjusted for age.

RFS: relapse-free survival, HR: Hazard ratio, CI: confidence interval, Gene overexpression: the expression level of an individual gene in tumor tissue normalized to adjacent non-tumor tissue from the same patient was categorized as “overexpression” by using the cutoff value determined from the ROC curve.

Bold face: statistical significant (P value < 0.05).

### Suppression of RUNX2 decreased miR-10a/b expression and impaired cell migration and invasion ability

**Figure 2:**
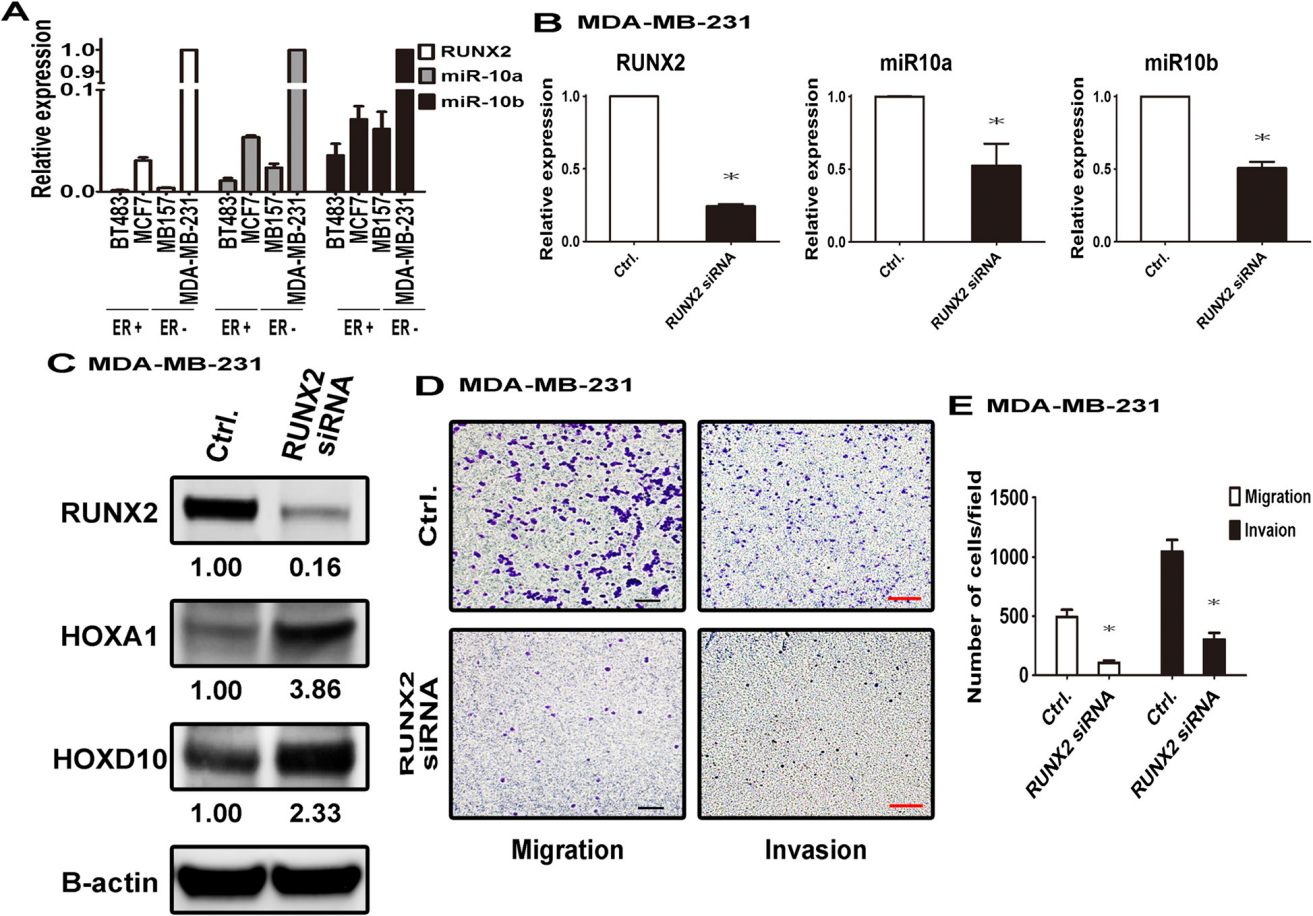
**RUNX2 silencing reduced the expression of miR-10a and miR-10b and inhibited cell migration and invasion. (A)** Expression level of miR-10a and miR-10b in breast cancer cell lines were analyzed by qPCR analysis and normalized to U6 snRNA expression. **(B)** MDA-MB-231 cells were transfected with 25 μM control siRNA or RUNX2 siRNA for 72 hours. RUNX2, miR-10a or miR-10b expression levels were determined by qPCR analysis. **(C)** Expression levels of RUNX2, HOXA1 and HOXD10 proteins were assessed by Western blot analysis and normalized to actin. **(D)** Cell migration and invasion ability were assessed by Transwell assay or Matrigel assay, respectively as described in Material and Methods. Black line: 20 μl, Red line: 100 μl **(E)** The quantification result was based on three independent experiments. *: *p* < 0.05, t-test.

In view of our findings of significant correlations among RUNX2 or miR-10a/b and relapse or death, we next investigated the inter-relationship of these 3 genes in breast cancer cell lines *in vitro*. Using qPCR analysis, we determined the expression levels of RUNX2 and miR-10a/b in 4 breast cancer cell lines. As shown in Figure 2A, MDA-MB-231, the invasive estrogen receptor negative (ER-) cell line, showed the highest expression levels of all these three molecules. Since MDA-MB-231 cells expressed very little Twist [31,32], which is reported to upregulate miR-10b [26] and since RUNX2 is involved in transcriptional activities of Twist [33], we explored the possible regulation of miR-10a/b by RUNX2. Silencing of RUNX2 by siRNA transfection of MDA-MB-231 cell line to 24% (p < 0.0001) of control resulted in a significant decrease in expression of miR-10a and miR-10b to 52% (p = 0.0349) and 51% (p < 0.0001), respectively (Figure 2B). Consistent with this, HOXA1 and HOXD10 are putative targets for miR-10a/b in Garzon *et al.* 2006 [34], suppression or overexpression of miR-10a/b increased or decreased HOXA1/HOXD10 protein expression, respectively, in breast cancer cell lines explained in Additional file 1: Figure S2, escalated in the silenced RUNX2 cells by 3.86 and 2.33 fold, respectively (Figure 2C). In line with the increased HOXA1 and HOXD10, migration and invasion ability of MDA-MB-231 cells were significantly reduced to 21% (p = 0.0035) and 29% (p = 0.0029), respectively. (Figure 2D, E). Thus, suppression of RUNX2 reduced miR-10a/b expression, and thereby impairing breast cancer cell motility.

### Overexpression of RUNX2 induced transcription of miR-10a/b and promoted cell migration and invasion

**Figure 3:**
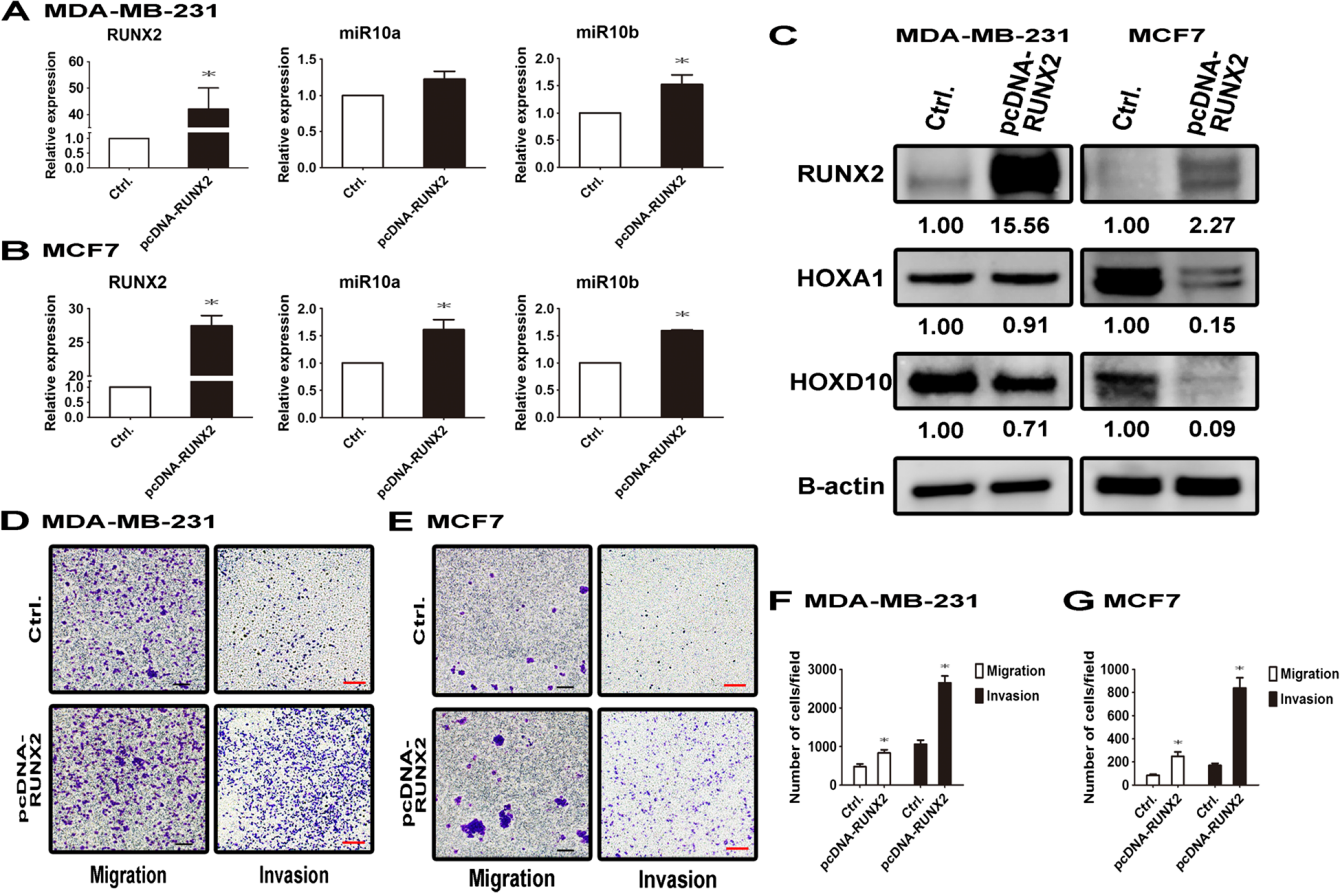
**Overexpression of RUNX2 induced expression of miR-10a and miR-10b and promoted cell migration and invasion. (A, B)** MDA-MB-231 or MCF7 cells were transfected with 4 μg control pcDNA3.1 vector or pcDNA-RUNX2 plasmid for 72 hours. RUNX2, miR-10a or miR-10b expression levels were analyzed by qPCR. **(C)** RUNX2, HOXA1 and HOXD10 protein levels were assessed by Western blot analysis and normalized to actin. **(D, E)** Cell migration and invasion ability were assessed by Transwell assay or Matrigel assay, respectively. Black line: 20 μl, Red line: 100 μl **(F, G)**. The quantification result was based on three independent experiments. *: *p* < 0.05, t-test.

In order to clarify the relationship between RUNX2 and miR-10a/b, RUNX2 was overexpressed in high and low RUNX2 expressing breast cancer cell lines, MDA-MB-231 and MCF7 (Figure 3A, B). RUNX2 increased the transcription of miR-10a by 1.61 fold (p = 0.0079) and that of miR-10b by 1.60 fold (p < 0.0001) in MCF7 cells and increased only the expression of miR-10b by 1.52 fold (p = 0.0413) in MDA-MB-231 cells. Consistently, protein levels of HOXA1 and HOXD10 markedly declined to 0.15 and 0.09 fold, respectively, in RUNX2 overexpressing MCF7 cells, but only slightly decreased to 0.91 and 0.71 fold in MDA-MB-231 cells (Figure 3C). Functional assay showed that cell migration and invasion were significantly enhanced in RUNX2 overexpressing MDA-MB-231 (1.74 fold, p = 0.0315 and 2.51 fold, p = 0.0016, for migration and invasion, respectively) and MCF7 cells (2.96 fold, p = 0.0143 and 4.95 fold, p = 0.0018, respectively) (Figure 3D, E, F, G). Thus, overexpression of RUNX2 upregulated miR-10a/b expression, thereby promoting breast cancer cell motility.

To sum up, these findings suggest that RUNX2 plays an important role in controlling breast cancer cell motility through regulation of miR-10a/b expression, and overexpression of these three genes has poor prognosis.

## Discussion

Metastasis is the major cause of mortality in cancer and affects the clinical progression of cancer patients [35]. In this study, primary breast tumor tissues were found to have higher expression levels of RUNX2 and miR-10a/b than adjacent non-tumor tissue, and overexpression in any of these three genes was significantly associated with poor clinical outcome.

Recently, Ma et al. has shown that the transcription factor Twist regulates the expression of miR-10b, which inhibits HOXD10 protein synthesis, thereby permitting the production of RHOC proteins and activating migration and invasion of breast cancer cells. Analyses of clinical specimens also indicate that miR-10b is highly expressed in not only metastatic breast cancer [26], but also early stage breast cancer [25]. However, as suggested in another study, in early-stage breast cancer patients, the expression of miR-10b appears to show no significant association with the development of distant metastases, recurrence-free survival, distant-relapse-free survival, or breast-cancer-specific survival [36]. In this study, we showed that RUNX2 upregulated miR-10a/b and promoted breast cancer cell migration and invasion. Furthermore, we demonstrated that miR-10a played a role in cell motility, similar to miR-10b [26], thus providing an additional mechanism in regulating breast cancer metastasis and invasion.

We found that, the expression of RUNX2 and miR-10a/b in ER- and triple negative breast cancer was higher than in ER + breast cancer. This observation may be related to the intricate interactions among estrogen, estrogen receptor alpha (ERα) and RUNX2, which is of crucial importance in osteoporosis and breast cancer. ERα can form a complex with RUNX2 to affect the capability of RUNX2 in transcribing downstream genes [37]. RUNX2 regulates the expression of ERα by interacting with its F promoter, one of the multiple promoters of the human ERα gene [33]. Also to be noted is that estrogen may also regulate the expression and activation of RUNX2 through various signal transduction pathways, notably the estrogen-transforming growth factor-beta (TGFβ) pathway [38], the Wnt pathway [39], the Fas/Fas Ligand pathway [40], and the nuclear factor-kappaB (NFκB) pathway [41]. Estrogen, ER and RUNX2 work to mutually regulate their expression and activation. This may help to explain the lower expression of RUNX2 in ER + breast cancer than ER- tumors.

We illustrated the regulation of the expression of miR-10a/b by RUNX2 in breast cancer cells. Several putative RUNX2 transcription factor binding sites were found at the proximal sequences preceding pre-miR-10a/b, based on website software PROMO analysis (http://alggen.lsi.upc.es/cgi-bin/promo_v3/promo/promoinit.cgi?dirDB=TF_8.3) [42-44]. Several different reporter constructs that carried putative RUNX2 binding sites within 15 k/24 k upstream of pre-miR-10a/b, respectively, were generated (Additional file 1: Table S5). We then compared the ability of RUNX2 to promote the transcription of reporter constructs carrying the RUNX2 sites. Unfortunately, RUNX2 failed to enhance the transcription of the miR-10a/b promoter regions that we cloned (Additional file 1: Figure S3). Studies to delineate the transcriptional regulation of miR-10a/b by RUNX2 will be further pursued in the future. Moreover, the RUNX2 cofactors, including STAT1, Twist, YAP or ERα protein [37,45], have been reported to play important roles in RUNX2 transcription ability and regulation of downstream gene expression. These RUNX2 cofactors will be incorporated into future studies to further clarify how RUNX2 regulates miR-10a/b transcription.

## Conclusion

Through *in vitro* experiments, our study demonstrates the impact of RUNX2 on breast cancer migration and invasion and its regulation of miR-10a/b expression. Importantly, our clinical-laboratory correlative analyses indicate that breast cancer patients with higher expression of RUNX2 and miR-10a/b – either individually or jointly – tend to suffer a greater risk of recurrence or death. Therefore, the three genes – RUNX2, miR-10a, and miR-10b – are deemed valuable markers for prognostication of breast cancer patients.

## Additional file

Additional file 1: Table S1. Correlative analysis of expression of miR-10a, miR-10b and RUNX2 with clinical parameters of breast cancer patients. **Table S2.** ROC analysis of expression of miR-10a, miR-10b, RUNX2 for predicting outcome in breast cancer patients. **Table S3.** The relationship between overexpression of individual gene or joint effects of miR-10a, miR-10b, and RUNX2 with prognosis in breast cancer patients. **Table S4.** Cox regression model analyses of the gene expression regarding OS of breast cancer patients. **Table S5.** Predicted RUNX2 binding sites at proximal sequences preceding pre- miR-10a and pre-miR-10b. **Figure S1.** Prognostic significance of increased expression levels of RUNX2, miR-10a or miR-10b in breast cancer patients. **Figure S2.** Target gene expression of suppression or overexpression miR-10a and miR-10b. **Figure S3.** Luciferase reporter assay of putative RUNX2 target genes.

## Abbreviations

RUNX2: Runt-related transcription factor 2
miR: miRNA, microRNA
ER: Estrogen receptor
PR: Progesterone receptor
SD: Standard deviation
ROC: Receiver operating characteristic
AUC: Areas under the curves
RFS: Relapse-free survival
OS: Overall survival
CI: Confidence interval
MEM: Modified eagle medium
FBS: Fetal bovine serum
DMEM: Dulbecco’s modified eagle medium
RPMI: Roswell Park Memorial Institute medium
ATCC: American Type Culture Collection
U6: RNU6B
Ct: Threshold cycle
SDS: Sodium dodecyl sulfate
PBS: Phosphate buffered saline
PBST: PBS containing 0.1% Tween20
OR: Odds ratio
HR: Hazard ratio
ERα: Estrogen receptor alpha
TGFβ: Transforming growth factor-beta
NFκB: Nuclear factor-kappaB
