# Supplementary material for: The prognostic significance of RUNX2 and miR-10a/10b and their inter-relationship in breast cancer
Source: J Transl Med. 2014 Sep 30;12:257. doi: 10.1186/s12967-014-0257-3 (PMC4189660; doi:10.1186/s12967-014-0257-3)
Supplement: Additional file 1: Table S1. — Correlative analysis of expression of miR-10a, miR-10b and RUNX2 with clinical parameters of breast cancer patients. Table S2. ROC analysis of expression of miR-10a, miR-10b, RUNX2 for predicting outcome in breast cancer patients. Table S3. The relationship between overexpression of individual gene or joint effects of miR-10a, miR-10b, and RUNX2 with prognosis in breast cancer patients. Table S4. Cox regression model analyses of the gene expression regarding OS of breast cancer patients. Table S5. Predicted RUNX2 binding sites at proximal sequences preceding pre- miR-10a and pre-miR-10b. Figure S1. Prognostic significance of increased expression levels of RUNX2, miR-10a or miR-10b in breast cancer patients. Figure S2. Target gene expression of suppression or overexpression miR-10a and miR-10b. Figure S3. Luciferase reporter assay of putative RUNX2 target genes. [file 12967_2014_257_MOESM1_ESM.pdf]

Supplementary data

Materials and methods

MiR-10a and miR-10b promoter region plasmid synthesis

Human genomic DNA (Promega, Madison, WI, USA) was used as the template for subcloning miR-10a/b reporter assay plasmid. The PCR primer pairs (miR-10a-p1-10409-forward:AAGGTACCTGAAAAGTATGAGCCTGGCTACAG;miR-10a-p1-12308-reverse:AAGCTAGCGACTTCTCGGCGATTTTTACGA;miR-10a-p2-6255-forware:AACCCGGGCACCCTGGTTTGAAGACATATCATC;miR-10a-p2-8794-reverse:AAAAGCTTGAGACGCCTAAATCACTGAGAAAAG;miR-10b-p1-16081-for:AAGGTACCAATGGGCTCAAATGTGTCAACA;miR-10b-p1-13182-reverse:AAGCTAGCGACAGCGACCCCCCACTT;miR-10b-p2-13068-forware:AAGAGCTCCGGGTAGCCACAGACAGGAT;miR-10b-p2-8965-reverse:AAGCTAGCTGTCGCTGGCTCTTGAACCT) were used. The amplified gDNAs of miR-10a-p1-10409-12308, miR-10a-p2-6255-8794, miR-10b-p1-16081-13182, miR-10b-p2-13068-8965 were then cloned into pGL3-basic vector (Promega) by ligation of the *KpnI- NheI, SmaI- HindIII, KpnI- NheI, SacI- NheI* fragments, respectively, to generate the pGL3-miR-10a-p1, pGL3-miR-10a-p2, pGL3-miR-10b-p1, pGL3-miR-10b-p2 constructs. The sequences of each plasmid were confirmed by DNA sequencing.

Luciferase reporter assay

$1.5X10^5$ 293T cells were plated into 12 well plate overnight before transfection. Two μg enhance plasmid (pcDNA3.1 or pcDNA-RUNX2), 0.5μg reporter plasmid (pGL3-basic, pGL3-miR-10a-p1, pGL3-miR-10a-p2, pGL3-miR-10b-p1, pGL3-miR-10b-

p2) and 0.05μg empty vector (phRGTK) plasmid were co-transfected with Lipofectamine 2000 (Invitrogen) for 24 hours. Then cells were lysed and luciferase assays were performed using a Dual-Luciferase® Reporter Assay System (Promega) on a single automatic injection Mithras (Berthold technologies, Bad Wildbad, German) luminometer following the manufacturer’s protocol. Transfection of each construct was performed in triplicate in each assay and a total of three assays were performed on three separate days. Luciferase readings were taken as singlets. Ratios of Firefly luciferase readings to Renilla luciferase readings were taken for each experiment and triplicates were averaged. The average values of the tested constructs were normalized to the activity of the empty construct. Histogram represented the averages of the normalized values with error bars indicating the range.

Supplementary Table S1 Correlative analysis of expression of miR-10a, miR-10b and RUNX2 with clinical parameters of breast cancer patients

	miR-10a				miR-10b				RUNX2			
**Variable**	**Mean ± SD**	**Median (Range)**	**P value[a]**	**P value[b]**	**Mean ± SD**	**Median (Range)**	**P value[a]**	**P value[b]**	**Mean ± SD**	**Median (Range)**	**P value[a]**	**P value[b]**
**Gender**												
**Male**	1.51	1.51		Ref.	3.94	3.94		Ref.	0.41	0.41		Ref.
**Female**	2.02±0.28	1.16 (0.01 ~ 18.57)		0.7975	3.13±0.42	1.71 (0.07 ~ 26.72)		0.3864	527.31±385.30	2.23 (0.01 ~ 37902.36)		0.1276
**Histology**												
**Ductal**	1.89±0.25	1.22 (0.01 ~ 13.55)	Ref.	Ref.	3.04±0.45	1.71 (0.07 ~ 26.72)	Ref.	Ref.	192.59±174.99	2.08 (0.26 ~ 16612.71)	Ref.	Ref.
**Other**	2.94±1.37	1.13 (0.07 ~ 18.57)	0.4654	0.6301	3.79±1.17	1.91 (0.37 ~ 15.79)	0.5586	0.2755	2932.87±2914.14	3.05 (0.01 ~ 37902.36)	0.3663	0.5394
**Grade**												
**I**	0.92±0.50	0.44 (0.07 ~ 3.84)	Ref.	Ref.	1.64±0.55	1.36 (0.16 ~ 4.35)	Ref.	Ref.	2.85±1.22	1.97 (0.26 ~ 9.71)	Ref.	Ref.
**II**	1.25±0.20	0.93 (0.01 ~ 4.86)	0.5069	0.3484	1.75±0.22	1.55 (0.08 ~ 6.54)	0.8371	0.7924	490.48±461.39	2.07 (0.41 ~ 16612.71)	0.2978	0.6809
**III**	2.61±0.44	1.51 (0.05 ~ 18.57)	**0.0210**	0.0629	4.16±0.67	2.05 (0.07 ~ 26.72)	**0.0068**	0.2995	614.59±601.43	2.50 (0.01 ~ 37902.36)	0.3130	0.4336
**Stage**												
**0**	1.69±0.41	1.58 (1.04 ~ 2.45)	Ref.	Ref.	5.37±3.71	2.46 (0.92 ~ 12.73)	Ref.	Ref.	3.28±2.17	1.42 (0.80 ~ 7.61)	Ref.	Ref.
**I**	1.76±0.41	0.85 (0.01 ~ 11.91)	0.9597	0.3585	2.98±0.70	1.65 (0.08 ~ 20.34)	0.3571	0.4198	32.27±27.99	2.03 (0.26 ~ 955.43)	0.3092	0.8023
**II**	2.18±0.50	1.29 (0.07 ~ 18.57)	0.8058	0.6021	3.19±0.70	1.57 (0.08 ~ 26.72)	0.4511	0.4403	6.88±2.80	2.15 (0.01 ~ 127.12)	0.7469	0.7544
**III**	2.14±0.54	1.37 (0.04 ~ 10.56)	0.7735	0.6713	3.01±0.78	1.77 (0.07 ~ 17.88)	0.3535	0.4635	2291.63±1695.37	2.84 (0.39 ~ 37902.36)	0.1902	0.7284
**Regional lymph node involvement**												
**No**	1.79±0.33	0.98 (0.01 ~ 13.55)	Ref.	Ref.	3.12±0.60	1.40 (0.08 ~ 26.72)	Ref.	Ref.	19.59±15.36	1.99 (0.26 ~ 955.43)	Ref.	Ref.
**Yes**	2.24±0.49	1.46 (0.04 ~ 18.57)	0.4352	0.1865	3.02±0.56	1.91 (0.07 ~ 17.88)	0.9068	0.3797	1253.59±932.02	2.70 (0.28 ~ 37902.36)	0.1926	0.4322
**PR**												
**Negative**	2.46±0.59	1.23 (0.04 ~ 18.57)	Ref.	Ref.	3.61±0.82	1.42 (0.08 ~ 26.72)	Ref.	Ref.	10.42±3.36	3.64 (0.54 ~ 127.12)	Ref.	Ref.
**Positive**	1.76±0.26	1.10 (0.01 ~ 11.91)	0.2852	0.7027	2.85±0.45	1.83 (0.07 ~ 20.34)	0.4192	0.8761	823.62±604.97	1.71 (0.01 ~ 37902.36)	0.1834	**0.0025**
**Her2**												
**Negative**	2.07±0.35	1.33 (0.04 ~ 13.55)	Ref.	Ref.	3.32±0.57	2.03 (0.11 ~ 26.72)	Ref.	Ref.	8.77±4.46	2.07 (0.01 ~ 233.94)	Ref.	Ref.
**Positive**	1.97±0.43	1.13 (0.01 ~ 18.57)	0.8513	0.3762	2.95±0.61	1.44 (0.07 ~ 20.34)	0.6523	0.0698	1017.42±746.84	2.23 (0.30 ~ 37902.36)	0.1825	0.6828

PR: progesterone receptor, HER2: HER2/neu, Ref.: reference group

The expression level of an individual gene in tumor tissue as determined by qPCR was normalized to that of adjacent non-tumor tissue from the same patient($2^{-\Delta\Delta Ct}$).

[a] P value for independent t-test

[b] P value for Wilcoxon rank-sum test

Bold face: statistical significant (P value<0.05)

Supplementary Table S2 ROC analysis of expression of miR-10a, miR-10b, RUNX2 for predicting outcome in breast cancer patients

	Gene overexpression (fold change; tumor vs. non-tumor)			
	**miR-10a**	**miR-10b**	**RUNX2**	**Combining three genes**
**Non-relapse vs. relapse**				
**AUC**	**0.7363**	**0.7756**	**0.6271**	**0.7713**
**95%CI**	0. 5806 ~ 0.8919	0.6526 ~ 0.8987	0.5058 ~ 0.7475	0.5675 ~ 0.9750
**Alive vs. death**				
**AUC**	0.5990	**0.6427**	0.6750	**0.7019**
**95%CI**	0.4893 ~ 0.7086	0.5345 ~ 0.7510	0.4272 ~ 0.9228	0.6072 ~ 0.7967
**No regional lymph node involvement vs. yes**				
**AUC**	0.5757	0.5013	0.5451	**0.6195**
**95%CI**	0.4651 ~ 0.6863	0.3392 ~ 0.5600	0.4290 ~ 0.6612	0.5154 ~ 0.7236

ROC: receiver operating characteristic, AUC: areas under the ROC curves, CI: confidence interval, Gene overexpression: the expression level of the individual genes in tumor tissue as determined by qPCR was normalized to that of adjacent non-tumor tissue of the same patient and was categorized as ‘‘overexpression’’ by using the cutoff value determined from the ROC curve

Bold face: statistical significant (P value<0.05)

Supplementary Table S3 The relationship between overexpression of individual gene or joint effects of miR-10a, miR-10b, and RUNX2 with prognosis in breast cancer patients

	Outcomes					
	Alive	Dead				
Variable	Number (%)	Number (%)	P-value	OR	95% CI	P value
miR-10a			0.1374			
≦1.35	57(96.6 %)	2(3.3 %)		1.00		
>1.35	43(87.8 %)	6(12.2 %)		3.57	0.68 ~ 18.82	0.1333
miR-10b			**0.0220**			
≦1.91	58(98.3 %)	1(1.7 %)		1.00		
>1.91	42(85.7 %)	7(14.3 %)		9.27	1.09 ~ 78.63	**0.0413**
RUNX2			0.0552			
≦3.81	72(96.0 %)	3(4.0 %)		1.00		
>3.81	28(84.8 %)	5(15.2 %)		4.34	0.96 ~ 19.63	0.0568
Number of overexpressing genes			**0.0030**			
0	37(97.4 %)	1(2.6 %)		1.00		
1	23(95.8 %)	1(4.2 %)		1.67	0.10 ~ 28.29	0.7222
2	30(96.8 %)	1(3.2 %)		1.18	0.07 ~ 19.83	0.9070
3	10(66.6 %)	5(33.3 %)		17.05	1.76 ~ 165.01	**0.0143**
Additive model of gene overexpression				2.90	1.22 ~ 6.89	**0.0159**

	Regional lymph node involvement					
	No	Yes				
Variable	Number (%)	Number (%)	P-value	OR	95% CI	P value
miR-10a			**0.0444**			
≦1.34	39(67.2 %)	19(32.8 %)		1.00		
>1.34	23(47.9 %)	25(52.1 %)		2.23	1.01 ~ 4.96	**0.0484**
miR-10b			0.3409			
≦1.73	34(63.0 %)	20(37.0 %)		1.00		
>1.73	28(53.8 %)	24(46.2 %)		1.45	0.67 ~ 3.15	0.3498
RUNX2			0.0913			
≦3.33	45(64.3 %)	25(35.7 %)		1.00		
>3.33	17(47.2 %)	19(52.8 %)		2.05	0.90 ~ 4.67	0.0868
Number of overexpressing genes			0.1716			
0	24(72.7 %)	9(27.3 %)		1.00		
1	15(60.0 %)	10(40.0 %)		1.81	0.59 ~ 5.53	0.3013
2	16(48.5 %)	17(51.5 %)		2.83	1.01 ~ 7.89	**0.0474**
3	7(46.7 %)	8(53.3 %)		3.04	0.85 ~ 10.84	0.0866
Additive model of gene overexpression				1.51	1.03 ~ 2.21	**0.0342**

OR: odds ratio, CI: confidence interval, Gene overexpression: the expression level of an individual gene in tumor tissue normalized to adjacent non-tumor tissue from the same patient was categorized as ‘‘overexpression’’ by using the cutoff value determined from the ROC curve

ORs and 95 % CIs were estimated in the logistic regression model, in which a group of dummy variables was used to represent different groups of patients showing different numbers of overexpressing genes.

Bold face: statistical significant (P value<0.05)

Supplementary Table S4 Cox regression model analyses of the gene expression regarding OS of breast cancer patients

	OS		
**Variable**	**HR**	**95% CI**	**P value**
**miR-10a>1.35 V.S miR-10a≦1.35**	3.66	0.72 ~ 18.54	0.1175
**miR-10b>1.91 V.S miR-10b≦1.91**	8.54	1.04 ~ 69.94	**0.0457**
**RUNX2>3.81 V.S RUNX2≦3.81**	3.91	0.93 ~ 16.37	0.0622
**Number of overexpressing genes**			
**0**	1.00		
**1**	1.65	0.10 ~ 26.41	0.7237
**2**	1.20	0.07 ~ 19.36	0.8974
**3**	14.59	1.66 ~ 128.24	**0.0157**
**Additive model of gene overexpression**	2.88	1.26 ~ 6.61	**0.0125**

Adjusted for age

OS: overall survival, HR: Hazard ratio, CI: confidence interval, Gene overexpression: the expression level of an individual gene in tumor tissue normalized to adjacent non-tumor tissue from the same patient was categorized as ‘‘overexpression’’ by using the cutoff value determined from the ROC curve

Bold face: statistical significant (P value<0.05)

Supplementary Table S5 Predicted RUNX2 binding sites at proximal sequences preceding pre- miR-10a and pre-miR-10b

RUNX2 binding sequence	MiR-10a	MiR-10b
TGTGGT	-43684, -43631, -37995, -27381, -23681, -21773, -12523, **-11967**, -10175, **-8695, -7901**	-39969, -34182, -33467, -30570, -30360, -30349, -26331, -25874, -23899, -18213, **-15800, -12030**
AACCACA	-34874, -21972, -20614, -20274	-5094
AACCGCA	-2533	-21273
GACCGCA	-28042, -16660	
GACCACA		-35013, -32316, -19267

Binding sites shown in bold face were selected for luciferase reporter assay

Supplementary Figure S1

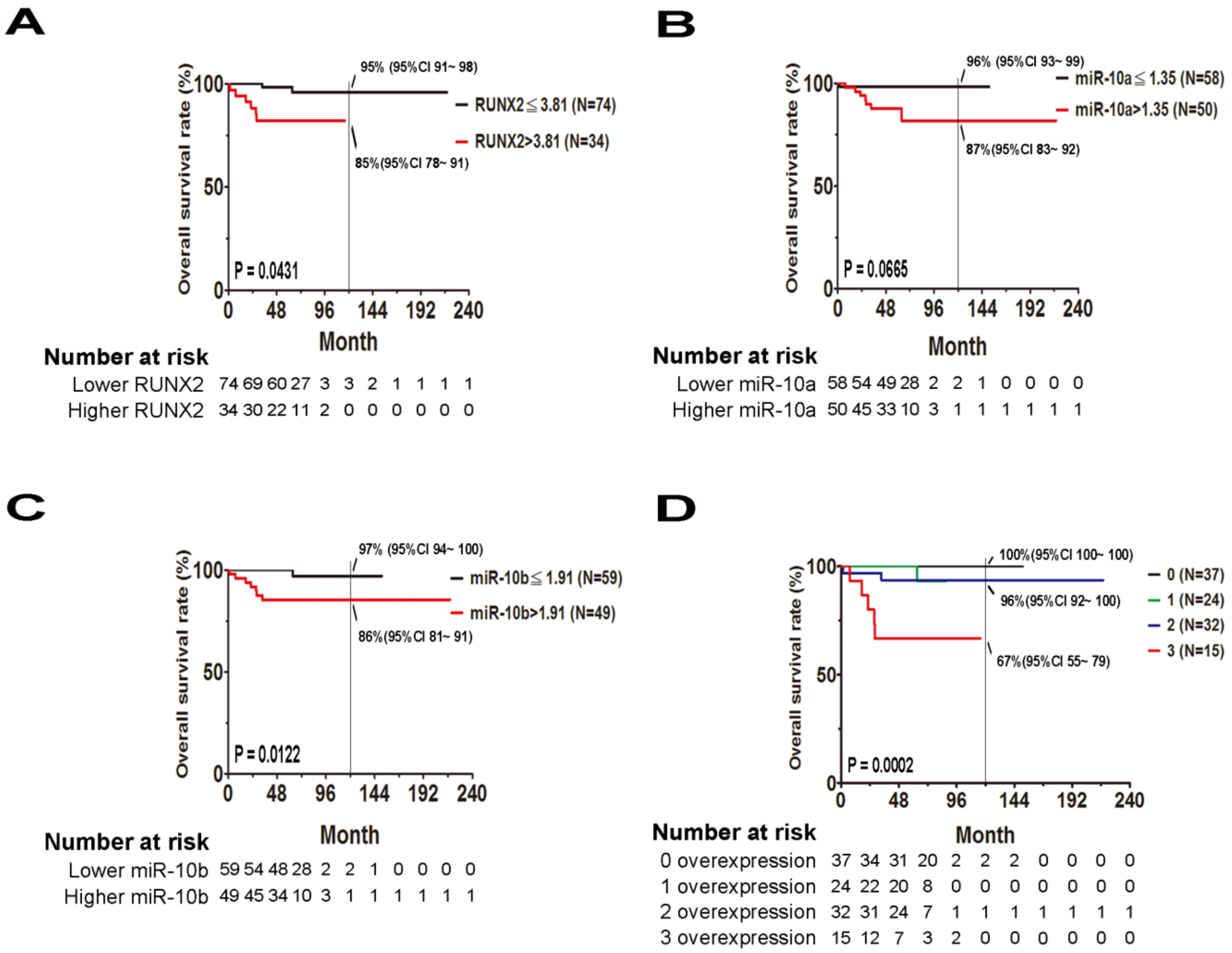

Supplementary Figure S1 Prognostic significance of increased expression levels of RUNX2, miR-10a or miR-10b in breast cancer patients.

Kaplan-Meier statistical analyses were conducted to examine the association between overall survival and the expression of RUNX2 (A), miR-10a (B) or miR-10b (C), and the number of overexpressed genes (D) in all patients.

Supplementary Figure S2

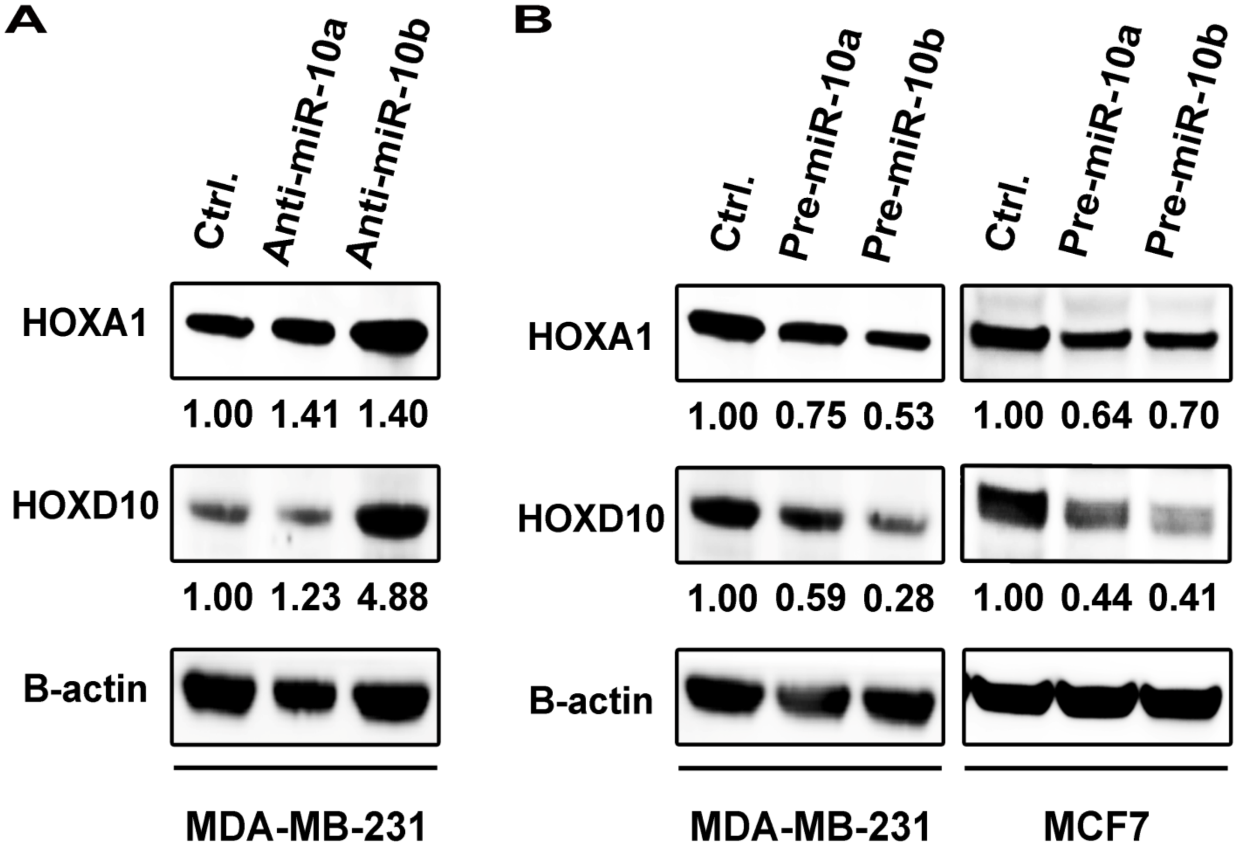

Supplementary Figure S2 Target gene expression of suppression or overexpression miR-10a and miR-10b

MDA-MB-231 cells were transfected with 50µM anti-miR control, anti-miR-10a or anti-miR-10b for 72hrs (A). MDA-MB-231 or MCF7 cells were transfected with 25µM pre-miR control, pre-miR-10a or pre-miR-10b for 72hrs (B). HOXA1 and HOXD10 protein expression level were assessed by Western blot analysis and normalized to actin protein level.

Supplementary Figure S3

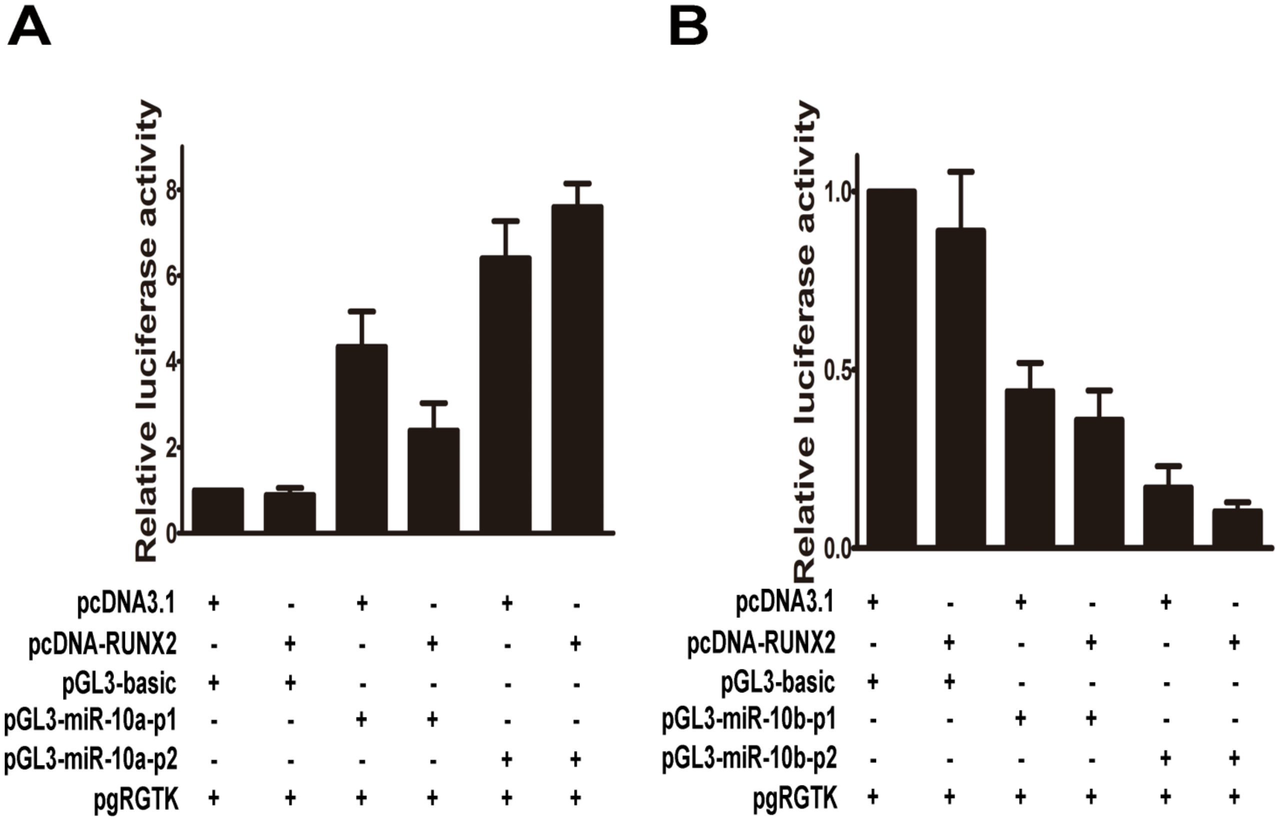

Supplementary Figure S3 Luciferase reporter assay of putative RUNX2 target genes
Human 293T cells were transfected with indicated plasmids for 24 hrs and cell lysates were collected for luciferase reporter assay. Firefly luciferase reporter gene expression from miR-10a (A) and miR-10b (B) was determined and data analyzed as described in “Materials and Methods”. Firefly luciferase was normalized by Renilla luciferase to correct for transfection efficiency. Histogram displayed the average ± SD values of triplicate determinations.
